# Late presentation of X-linked inhibitor of apoptosis (XIAP) deficiency in a young adult

**DOI:** 10.1186/s13223-025-00965-4

**Published:** 2025-04-29

**Authors:** Samina Nazarali, Beata Derfalvi, Pascale Clark

**Affiliations:** 1 Department of Medicine, Division of Clinical Immunology and Allergy, Western University, London, ON Canada; 2 Division of Immunology, Department of Paediatrics, Dalhousie University, IWK Health, Halifax, NS Canada; 3 Division of General Internal Medicine, Dalhousie University, Halifax, NS Canada

**Keywords:** Inborn error of immunity, Stem cell transplant, HLH, XIAP

## Abstract

**Background:**

X-linked inhibitor of apoptosis (XIAP) deficiency is a rare inborn error of immunity which occurs secondary to mutations in the XIAP/BIRC4 gene. Disease onset usually manifests within the first few years of life, and is associated with a spectrum of clinical features, secondary to immune dysregulation. Males typically present with refractory chronic colitis, hemophagocytic lymphohistiocytosis, and severe and/or recurrent infections. Laboratory analysis may reveal hypogammaglobulinemia and cytopenias. At present, the only curative treatment is allogenic hematopoietic stem cell transplantation.

**Case presentation:**

A 24-year-old gentleman, immigrant from the Democratic Republic of Congo, was referred to outpatient immunology for evaluation of an inborn error of immunity given a past medical history significant for refractory fistulizing Crohn’s disease, arthritis, liver abscesses, prior disseminated tuberculosis, anemia, and recurrent infections. He had been asymptomatic throughout his childhood and adolescence, with no infections or symptoms of inflammatory disease until the age of 19, when he was diagnosed with Crohn’s disease. He was soon after admitted to hospital and was diagnosed with hemophagocytic lymphohistiocytosis. Primary immunodeficiency gene panel testing revealed a nonsense variant XIAP c833C > G p.(Ser278*), which generates a premature stop codon at exon 2 (of total 7 exons). On flow cytometry analysis, XIAP protein expression was significantly reduced, confirming the diagnosis of XIAP deficiency.

**Conclusion:**

This is one of the only documented reports of a patient with XIAP deficiency, presenting with symptom-onset in adulthood. This case highlights the need to maintain a high index of suspicion for XIAP deficiency in patients with the appropriate clinical presentation, despite advanced age of presentation.

## Background

X-linked inhibitor of apoptosis (XIAP) deficiency is a rare inborn error of immunity (IEI) which occurs secondary to mutations in the *XIAP/BIRC4* gene [2]. The XIAP protein belongs to a family of apoptotic suppressors and has anti-apoptotic effects through the inhibition of caspases 3, 7, and 9 [3]. As a result, T cells from patients with XIAP deficiency, more specifically invariant natural killer T cells and mucosal-associated invariant T cells, have increased sensitivity to activation-induced cell death [2]. Apart from its anti-apoptotic effects, XIAP is implicated in several immune pathways which have previously been described [2–4]. Its role in innate immunity comes from the finding that XIAP is required for nucleotide-binding oligomerization domain-containing protein-1 and − 2 receptor mediated nuclear factor κB signalling [5, 6]. Additionally, through its role in dectin-1 signalling, XIAP is implicated in anti-fungal immunity [7]. XIAP is also involved in regulating the activation of the NOD-, LRR-, and pyrin domain-containing protein (NLRP3) inflammasome, and XIAP loss results in dysregulation of caspase-1/NLRP3 inflammasome activation, overproduction of pro-inflammatory cytokines and cell death [4, 8]. Given that XIAP has been implicated in several immune pathways and that it is important for both pathogen clearance and regulation of the inflammatory response [4], patients with XIAP deficiency exhibit a range of clinical features.

XIAP deficiency was historically termed as a type of X-linked lymphoproliferative (XLP) syndrome, as it was believed to be phenotypically similar to signalling lymphocytic activation molecule deficiency or XLP-1. However, subsequent observations found that several clinical features differ between the two entities, more specifically, that XIAP deficient patients do not develop lymphomas, and over 80% develop hemophagocytic lymphohistiocytosis (HLH), classifying XIAP deficiency as part of a familial HLH syndrome [4]. It is estimated to occur in 1–2 million live male births; however, its true prevalence is likely higher [4, 6]. Disease onset usually manifests within the first few years of life, and is associated with a spectrum of clinical features, secondary to immune dysregulation. Males typically present with HLH, refractory chronic colitis, and severe and/or recurrent infections [9]. Laboratory analysis may reveal hypogammaglobulinemia and cytopenias [2, 9]. At present, the only curative treatment is allogenic hematopoietic stem cell transplantation (HSCT) [4].

## Case presentation

A 24-year-old gentleman, immigrant from the Democratic Republic of Congo, was referred to outpatient immunology for evaluation of an IEI given past medical history significant for refractory fistulizing Crohn’s, arthritis, liver abscesses, anemia, recurrent biopsy-proven dermatophyte infections affecting skin and lymph nodes positive for trichophyton violaceum requiring prolonged treatment with oral terbinafine, malaria, and prior schistosomiasis. Additionally, he was treated for latent tuberculosis (TB) and developed fulminant TB in the context of tumor necrosis factor inhibitor therapy, initially treated with ethambutol, isoniazid, moxifloxacin and pyrazinamide. Bronchoscopy confirmed pan-sensitive mycobacterium, and he completed a 10-month course of rifampin and isoniazid. Interestingly, he had been asymptomatic in childhood and adolescence, with no infections or symptoms of inflammatory disease, until the age of 19, when he developed severe abdominal pain and was diagnosed with Crohn’s disease. He failed multiple lines of treatment such as azathioprine, methotrexate, and multiple biologic agents (infliximab, adalimumab and vedolizumab). He required repeat courses of systemic steroids and extensive abdominal surgeries.

As he was being investigated as an outpatient, he was admitted to hospital with decreased oral intake, high ostomy output, fevers, and general malaise. He was treated with methylprednisolone for a suspected Crohn’s disease flare; however, after every attempt at a wean, he developed recurrent fevers. Imaging showed evidence of hepatosplenomegaly, hepatic and splenic lesions, and intra-abdominal and para-aortic lymphadenopathy. He became severely underweight, appeared emaciated and required total parenteral nutrition. He was initiated on broad spectrum antibiotics including caspofungin for possible hepatosplenic candidiasis.

At that time, primary immunodeficiency (PID) gene panel testing ordered by his outpatient clinical immunologist was reported and revealed a hemizygous nonsense variant XIAP c833C > G p.(Ser278*), which generates a premature stop codon at exon 2 (of total 7 exons). On flow cytometry analysis, XIAP protein expression was significantly reduced; however, sample integrity was considered poor.

He was soon after diagnosed with HLH, with a ferritin over 200,000 mcg/L, elevated soluble CD25 (> 27,000 pg/mL), pancytopenia with profound neutropenia, and bone marrow biopsy and aspirate showing scant hemophagocytes. Testing for cytomegalovirus was negative, and although Epstein Barr virus (EBV) polymerase chain reaction (PCR) was negative, EBV nuclear antigen serology was positive, indicating past EBV infection. Supplementation with intravenous immune globulin (IVIG) was initiated for hypogammaglobulinemia thought to be secondary to enteropathy (IgG 6.09 g/L, albumin 27 g/L). To evaluate for underlying lymphoma, positron emission tomography was completed, and was negative for malignancy, but noted progressive lung nodules. Given his past-medical history of disseminated TB that was previously treated, a bronchoscopy was done and revealed acid-fast bacilli, with subsequent PCR confirming TB. He was started on treatment for presumed multi-drug-resistant TB and was on rifampin, pyrazinamide, moxifloxacin, linezolid, meropenem, amoxicillin-clavulanic acid, and isoniazid. Complete HLH treatment, as per the HLH 2004 protocol was delayed due to active infection; however, he continued to deteriorate and soon received the complete regimen (etoposide, cyclosporine, and dexamethasone) and displayed partial response. The patient was transferred to a peripheral hospital once he was clinically stable. He was started on an interleukin-1 receptor antagonist, with further reduction in his biochemical markers and marked clinical improvement (Table 1).

**Table 1:** Lab values for the patient prior to treatment, after 8 weeks of HLH treatment, and post Anakinra initiation. N/A = not available. Bolded lab values indicate abnormal lab results outside the reference range

Lab indices	Prior to treatment	After 8 weeks of HLH treatment	Post anakinra initiation
**White blood cells** (4.5–11 × 10^9^/L)	**2.78 × 10** ^**9**^ **/L**	**0.53 × 10** ^**9**^ **/L**	**3.30 × 10** ^**9**^ **/L**
**Hemoglobin** (140–180 g/L)	**67 g/L**	**79 g/L**	**122 g/L**
**Platelets** (150–350 × 10^9^/L)	**54 × 10** ^**9**^ **/L**	**23 × 10** ^**9**^ **/L**	169 × 10^9^/L
**Ferritin** (22–300 mcg)	**> 200**,**000 mcg**	**38**,**786.2 mcg**	**824 mcg**
**Triglycerides** (< 1.70 mmol/L)	**4.70 mmol/L**	**2.05 mmol/L**	1.70 mmol/L
**Soluble CD25** (532–1891 pg/mL)	**> 27**,**000 pg/mL**	**2027 pg/mL**	N/A
**Lactate dehydrogenase** (120–230 U/L)	**3072 U/L**	**214 U/L**	**253 U/L**
**Fibrinogen** (2–4 g/L)	2.10 g/L	2.05 g/L	**4.98 g/L**

At his last follow up, he was doing remarkably well on anakinra (interleukin-1 receptor antagonist) and markers of HLH remained stable. He no longer required immunosuppressive therapy for IBD or IVIG, as his IgG levels remained at the upper limit of normal (last IgG 14.03 g/L). He had gained over 20 kg. His mother was found to be a carrier of the mutation, but his sister and two brothers did not inherit it. He was evaluated for HSCT, but recommendation was made against transplant, partly because only a haploidentical match was identified. He feels validated to have a diagnosis that explains his symptoms and hopes to soon be well enough to return to work.

## Discussion and conclusions

XIAP deficiency is an IEI caused by mutations in the *XIAP* gene, and to date, over 90 disease causing mutations have been identified with no genotype-phenotype correlation [4]. To our knowledge, our patient’s mutation has not yet been reported. These individuals, as highlighted in this case, are susceptible to recurrent infections, refractory IBD, and HLH. The monogenic form of IBD in these patients, is known to be distinct from adult Crohn’s disease in both its etiology and responsiveness to therapy [10]. Given the refractory nature of this patient’s Crohn’s disease and extensive history of infections, there were indications of a potentially dysregulated immune system.

Although normally diagnosed earlier in life, the late presentation of XIAP deficiency in this case demonstrates the heterogeneity of this condition. Previous reports have found that neither the type of mutation or residual protein expression is related to clinical presentation and it is hypothesized that there are likely other genetic and environmental factors that contribute to the overall phenotype [4, 11]. Additionally, limited access to healthcare may have delayed diagnosis in this case. Overall, XIAP deficiency is likely underdiagnosed but as genetic testing is becoming more readily accessible, new cases are being identified. A recent study reviewing reported cases of XIAP deficiency highlighted its evolving clinical picture and emphasizes the importance of recognizing the need for XIAP deficiency testing across clinical specialities and patient age ranges, as demonstrated in this case [12].

Early detection of XIAP deficiency allows for family counselling and timely screening of relatives. Additionally, the importance of early diagnosis comes from the finding that by the age of 20, 80% of patients develop HLH, which has a mortality rate of over 50% [13], and in XIAP deficiency, is noted to be recurrent. With regards to treatment, HSCT should be considered early in the disease course as it has been shown to treat intestinal inflammation and decrease risk of developing HLH; however, measures should be taken to prevent acute GVHD in these patients as they may be at higher risk of increased GVHD severity and mortality [14]. There also remains lack of data regarding overall benefit of HSCT later in the disease course [12]. As such, early detection is crucial, and further studies are required to elucidate disease progression and to drive new therapeutic approaches to improve the overall quality of life and patient outcomes.

To our knowledge, this is one of the first case reports of a XIAP deficient patient developing symptoms in adulthood. It underlines the need to maintain a high index of suspicion for XIAP deficiency in patients with the appropriate clinical presentation, despite advanced age of presentation.

## Data Availability

No datasets were generated or analysed during the current study.

## Abbreviations

EBV: Epstein Barr virus
EBNA: Epstein Barr virus nuclear antigen
HLH: Hemophagocytic lymphohistiocytosis
HSCT: Hematopoietic stem cell transplantation
IBD: Inflammatory bowel disease
IEI: Inborn error of immunity
IVIG: Intravenous immune globulin
NLRP3: NOD-, LRR-, and pyrin domain-containing protein 3
PCR: Polymerase chain reaction
PID: Primary immunodeficiency
TB: Tuberculosis
XIAP: X-linked inhibitor of apoptosis
